# Supplementary material for: Comprehensive Annotation and Functional Exploration of MicroRNAs in Lettuce
Source: Front Plant Sci. 2021 Dec 24;12:781836. doi: 10.3389/fpls.2021.781836 (PMC8739914; doi:10.3389/fpls.2021.781836)
Supplement: Supplementary file 2 [file Data_Sheet_8.ZIP › GSM2230749.plot/Lsa-miRN46_Lsat_1_v5_gn_9_32101.1_131_TPlot.pdf]

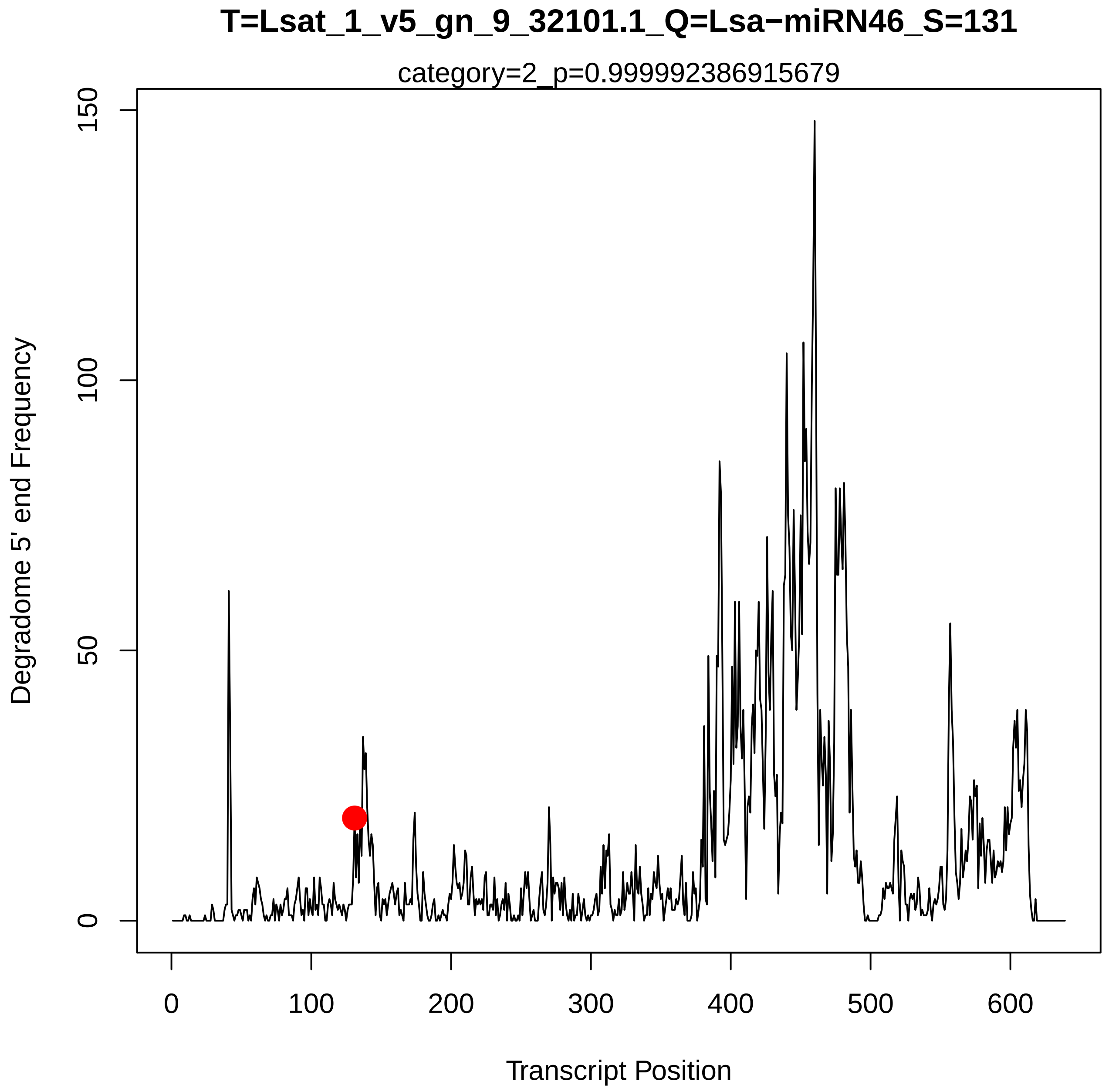
T=Lsat_1_v5_gn_9_32101.1_Q=Lsa−miRN46_S=131
category=2_p=0.999992386915679
Degradome 5' end Frequency
0
50
100
150
0
100
200
300
400
500
600
Transcript Position
